# Breaking the selectivity-uptake trade-off of photoimmunoconjugates with nanoliposomal irinotecan for synergistic multi-tier cancer targeting

**DOI:** 10.1186/s12951-019-0560-5

**Published:** 2020-01-02

**Authors:** Barry J. Liang, Michael Pigula, Yan Baglo, Daniel Najafali, Tayyaba Hasan, Huang-Chiao Huang

**Affiliations:** 1 Fischell Department of Bioengineering, University of Maryland, 8278 Paint Branch Drive, College Park, MD 20742 USA; 2 Wellman Center for Photomedicine, Massachusetts General Hospital and Harvard Medical School, Boston, MA 02114 USA; 3 Division of Health Sciences and Technology, Harvard University and Massachusetts Institute of Technology, Cambridge, MA 02139 USA; 4 Marlene and Stewart Greenebaum Cancer Center, University of Maryland School of Medicine, Baltimore, MD 21201 USA

**Keywords:** Photoimmunoconjugate, Irinotecan liposome injection, Benzoporphyrin derivative, Epidermal growth factor receptor, Multi-drug delivery

## Abstract

**Background:**

Photoimmunotherapy involves targeted delivery of photosensitizers via an antibody conjugate (i.e., photoimmunoconjugate, PIC) followed by light activation for selective tumor killing. The trade-off between PIC selectivity and PIC uptake is a major drawback limiting the efficacy of photoimmunotherapy. Despite ample evidence showing that photoimmunotherapy is most effective when combined with chemotherapy, the design of nanocarriers to co-deliver PICs and chemotherapy drugs remains an unmet need. To overcome these challenges, we developed a novel photoimmunoconjugate-nanoliposome (PIC-Nal) comprising of three clinically used agents: anti-epidermal growth factor receptor (anti-EGFR) monoclonal antibody cetuximab (Cet), benzoporphyrin derivative (BPD) photosensitizer, and irinotecan (IRI) chemotherapy.

**Results:**

The BPD photosensitizers were first tethered to Cet at a molar ratio of 6:1 using carbodiimide chemistry to form PICs. Conjugation of PICs onto nanoliposome irinotecan (Nal–IRI) was facilitated by copper-free click chemistry, which resulted in monodispersed PIC–Nal–IRI with an average size of 158.8 ± 15.6 nm. PIC–Nal–IRI is highly selective against EGFR-overexpressing epithelial ovarian cancer cells with 2- to 6-fold less accumulation in low EGFR expressing cells. Successful coupling of PIC onto Nal–IRI enhanced PIC uptake and photoimmunotherapy efficacy by up to 30% in OVCAR-5 cells. Furthermore, PIC–Nal–IRI synergistically reduced cancer viability via a unique three-way mechanism (i.e., EGFR downregulation, mitochondrial depolarization, and DNA damage).

**Conclusion:**

It is increasingly evident that the most effective therapies for cancer will involve combination treatments that target multiple non-overlapping pathways while minimizing side effects. Nanotechnology combined with photochemistry provides a unique opportunity to simultaneously deliver and activate multiple drugs that target all major regions of a cancer cell—plasma membrane, cytoplasm, and nucleus. PIC–Nal–IRI offers a promising strategy to overcome the selectivity-uptake trade-off, improve photoimmunotherapy efficacy, and enable multi-tier cancer targeting. Controllable drug compartmentalization, easy surface modification, and high clinical relevance collectively make PIC–Nal–IRI extremely valuable and merits further investigations in living animals.

## Background

Photoimmunotherapy (PIT) employs antibody-photosensitizer conjugates (i.e., photoimmunoconjugates, PICs) and harmless near-infrared light (λ = 600–900 nm) to induce reactive oxygen species (ROS)-mediated (e.g., ^1^O_2_, O_2_^•–^, •OH) tumor destruction while sparing normal tissues [1–4]. The fluorescence signal generated from the excited photosensitizers can be used for optical imaging and fluorescence-guided surgery (FGS) of tumors [5]. Epidermal growth factor receptor (EGFR) has long represented an oncologic target of immense interest, and it is overexpressed in several malignancies, including head and neck cancer, ovarian cancer, and glioblastoma [6]. Since the introduction of PIT in the ‘80s [4], several EGFR-targeted PICs (e.g., cetuximab-IRDeye700 and panitumumab-IRDye800) are now in clinical trials for PIT or FGS (NCT02422979, NCT03384238). We previously developed a PIC system that comprises of the U.S. Food and Drug Administration (FDA)-approved anti-EGFR monoclonal antibody cetuximab (Cet) and a clinically used benzoporphyrin derivative (BPD) photosensitizer to target cancer cells [7–11]. The highly self-quenched BPD molecules conjugated to Cet can be de-quenched (activated) by cancer cells via lysosomal proteolysis of the antibody [7, 10, 11]. It is also well-established that light activation of BPD induces photochemical disruption of the mitochondrial membrane [9], which triggers the release of cytochrome *c*, a potent initiator of apoptotic cell death [12–14]. This shifts the balance in the target cells from an anti-apoptotic state to a more pro-apoptotic state, mediating eventual cell death.

While PIT leverages PIC to minimize damage to healthy tissues, it requires an optimal intracellular PIC concentration threshold for effective tumor destruction [9, 11]. One of the strategies to overcome the insufficient PIC uptake is to combine nanotechnology with PIC. With a high surface area-to-volume ratio, nanoparticles can be decorated with large amounts of antibodies for tumor targeting [15]. We recently demonstrated that immobilization of PIC onto poly(lactic-co-glycolic acid) (PLGA) nanoparticles could facilitate the indirect endocytosis of high payloads of PIC under limited antibody-receptor binding events, overcoming the persistent challenge of insufficient PIC uptake by cancer cells [10]. However, it remains unclear if this ‘carrier effect’ phenomenon with PIC and PLGA nanoparticles could be extended to other types of nanoformulations at large. In this study, we seek to verify the generalizability of this phenomenon using a novel photoimmunoconjugate-nanoliposome (PIC–Nal) formulation. Furthermore, the PIC–Nal is rationally designed to co-deliver irinotecan chemotherapy for enhanced PIT outcomes.

Nanoliposomal irinotecan injection (Onivyde®, Nal–IRI) is a valuable chemotherapy given in combination with fluorouracil and leucovorin to patients with gemcitabine-refractory metastatic pancreatic cancer, and it is now being tested in patients with gastric adenocarcinoma (NCT03739801), gynecological cancer (NCT01770353), lung cancer (NCT03088813), and glioblastoma (NCT03119064) [16–19]. Irinotecan acts by inhibiting topoisomerase I (Top1) and trapping Top1-DNA cleavage complexes (Top1cc) to induce double-stranded DNA breaks in the nucleus and promote direct cell death [20]. We have shown that light activation of BPD (i.e., photodynamic therapy, PDT) synergizes with irinotecan to improve survival outcomes in pancreatic cancer mouse models [21–23]. Similarly, others also demonstrated that light activation of irinotecan-loaded porphysomes reduces pancreatic tumor burden [24]. However, all these studies utilized ‘non-targeted’ nanoliposomes carrying ‘unquenched’ photosensitizers that are at a higher risk of normal tissue phototoxicity. Here, we leverage our ‘tumor-activatable’ PIC system (i.e., Cet-BPD) [7, 9] to improve the selectivity and efficacy of irinotecan.

For many combinations to achieve optimal efficacy, spatiotemporal control of drug exposure to coordinate targeted inhibition of interconnected cancer survival and growth pathways is of paramount importance [25, 26]. In addition to targeting multiple survival pathways or blocking cell death escape mechanisms, drugs that are the best candidates for combination are those that target all major regions of a cell (i.e., plasma membrane, cytoplasm, and nucleus) and also have non-overlapping toxicities [27, 28]. Hybrid nanocarriers, such as those based on PICs and nanoliposomes, are particularly promising approaches for combination therapies because they can be designed to compartmentalize multiple agents at a fixed ratio, target deliver therapeutics to cancer cells at a high payload, and generate cytotoxic ROS upon light activation [29]. Here, we interface PIC and nanoliposomal irinotecan for targeted photoimmuno-chemotherapy. We anticipate the mechanism-based nanotechnology comprising Cet, BPD, and irinotecan will be more effective in reducing cancer viability by targeting different subcellular components as well as molecular pathways, compared to monotherapies. The following studies demonstrate how photoimmuno-chemotherapy addresses one of the major challenges facing PIT (i.e., PIC uptake) and provides compelling evidence that cooperative targeting EGFR, mitochondrial, and DNA can markedly improve treatment efficacy against cancer.

## Results

### Synthesis and characterization of PIC–Nal and PIC–Nal–IRI

**Fig. 1:**
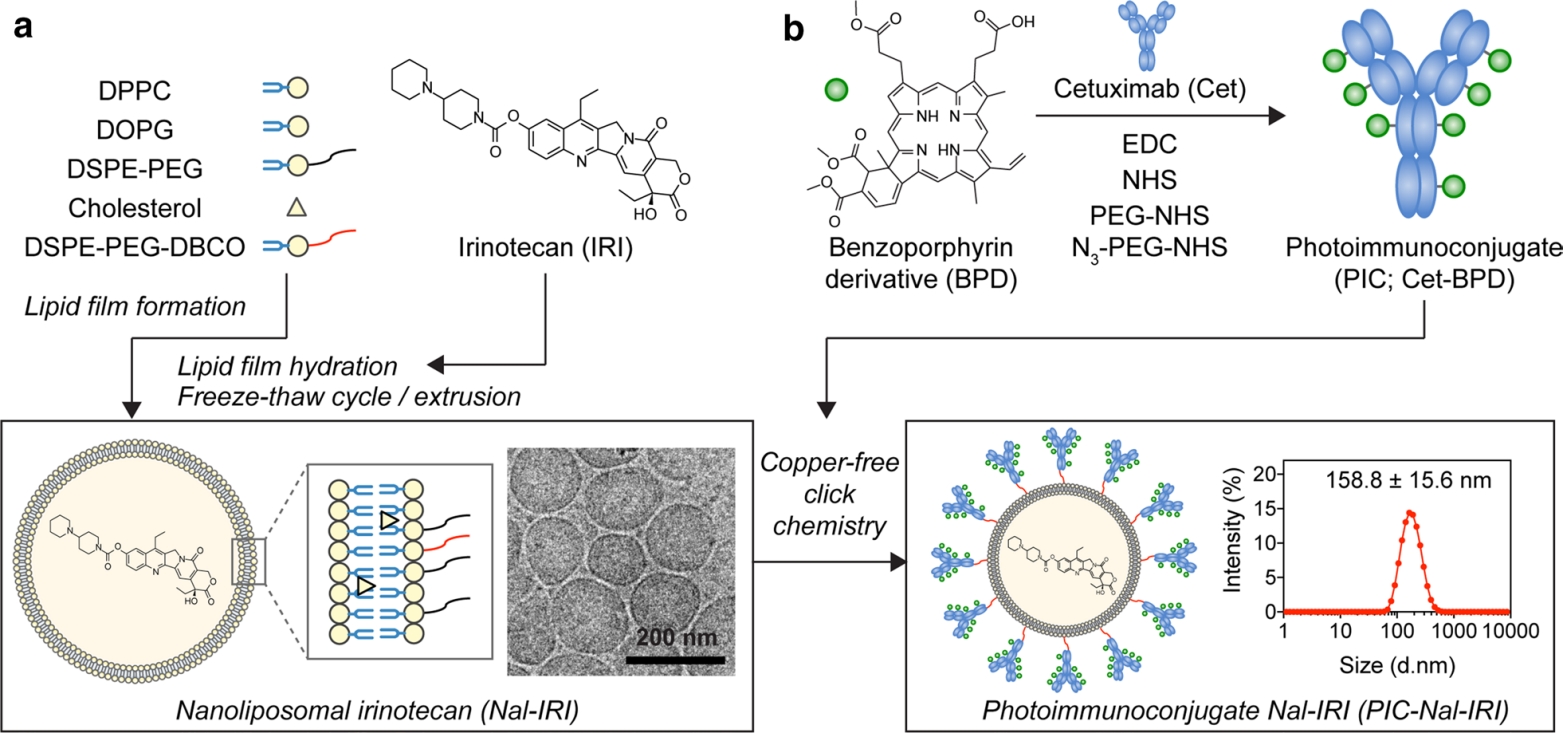
Schematic diagram of the steps for photoimmunoconjugate nanoliposomal irinotecan (PIC–Nal–IRI) synthesis. **a** Synthesis and cryogenic electron microscopy image of monodispersed nanoliposomal irinotecan (Nal–IRI) with an average size of ~ 150 nm (PdI < 0.1, *n* > 3). **b** Benzoporphyrin derivative (BPD) was covalently conjugated onto cetuximab (Cet) via carbodiimide chemistry to form photoimmunoconjugate (PIC). Copper-free click chemistry was employed to tether PICs onto Nal–IRI to form PIC–Nal–IRI with an average size of 158.8 ± 15.6 nm (PdI < 0.1, *n* > 3)

**Fig. 2:**
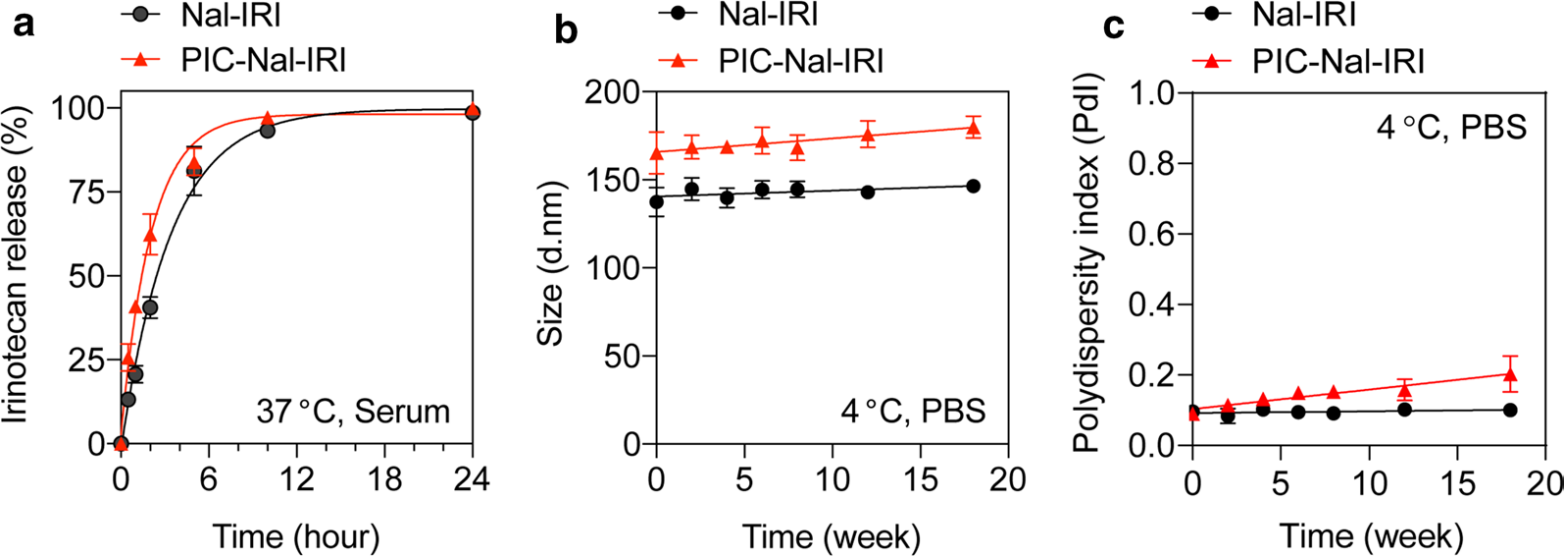
Drug release and the stability of Nal–IRI and PIC–Nal–IRI. **a** Both Nal–IRI and PIC–Nal–IRI exhibited similar irinotecan release profiles in serum-containing medium at 37 °C. **b**, **c** The long-term stability of Nal–IRI and PIC–Nal–IRI (4 °C, PBS) in dark was assessed by longitudinal monitoring of changes in **b** hydrodynamic size and **c** polydispersity index

Unilaminar nanoliposome (Nal) and nanoliposomal irinotecan (Nal–IRI), prepared via freeze–thaw cycle method, are 126.5 ± 3.5 nm and 151.0 ± 11.7 nm in diameter, respectively with a narrow size distribution (Polydispersity index, PdI < 0.1) (Fig. 1a; Table 1). To minimize the non-specific electrostatic interactions with the cell membrane and to maximize the contribution of specific interactions to binding and internalization [30, 31], the surface charge of nanoformulations was engineered to be neutral-to-slightly negative (between − 13.6 mV and − 19.6 mV; Table 1) by incorporating 6.9 mol% of dioleoylglycerophosphoglycerol (DOPG) into the lipid composition. To prepare PIC, BPD molecules were conjugated to Cet using carbodiimide chemistry (Fig. 1b). Overnight reaction of BPD-N-hydroxysuccinimide ester and Cet at 3:1, 6:1, and 9:1 molar ratios resulted in the formation of PICs with ~ 2, 4, and 6 BPD molecules per Cet, respectively. This corresponds to ~ 67% conjugation efficiency (Additional file 1: Table S1). Click chemistry conjugation of azide-functionalized PICs to DBCO-containing Nal or DBCO-containing Nal–IRI resulted in the formation of PIC–Nal and PIC–Nal–IRI with diameters of 142.5 ± 5.9 nm and 158.8 ± 15.6 nm, respectively (PdI < 0.1) (Table 1). The conjugation efficiency of PIC to Nal was ~ 66% (Table 1), which corresponds to ~ 40 PICs per Nal. Increasing the BPD-to-Cet ratio of PIC did not significantly alter the size, surface charge, or conjugation efficiency of the PIC–Nal (Additional file 1: Table S2). Irinotecan was passively encapsulated in the aqueous core of Nal and PIC–Nal at encapsulation efficiencies of 38.8 ± 4.4% and 23.7 ± 2.2%, respectively. The conjugation efficiency of PIC to Nal–IRI was 48.0 ± 2.7%, which corresponded to ~ 33 PICs per Nal–IRI. Drug release profiles of Nal–IRI and PIC–Nal–IRI were examined in human serum-containing medium at 37 °C (Fig. 2a). At 1 h post-incubation, we observed ~ 20% and ~ 42% release of irinotecan from the Nal–IRI and PIC–Nal–IRI, respectively. The relatively fast irinotecan release from PIC–Nal–IRI (*t*_1/2_ = 2 h) compared to Nal–IRI (*t*_1/2_ = 2.3 h) is likely due to the presence of PIC, suggesting that irinotecan will be readily available to the cancers cells when PIT occurs. Stability studies showed that 4-month dark storage at 4 °C did not significantly alter the overall size and monodispersity of Nal–IRI and PIC–Nal–IRI (Fig. 2b, c).

**Table 1:** Physical characterization of the nanoformulations

Sample	Size (d. nm)	Polydispersity index (PdI)	Zeta potential (mV)	Irinotecan encapsulation efficiency (%)^a^	PIC conjugation efficiency (%)^b^	Number of PIC per Nal
Nal	126.5 ± 3.5	0.08 ± 0.01	− 19.6 ± 0.7	N/A	N/A	N/A
PIC–Nal	142.5 ± 5.9	0.06 ± 0.01	− 13.6 ± 0.6	N/A	66.5 ± 2.3	39.9 ± 1.4
Nal–IRI	151.0 ± 11.7	0.08 ± 0.01	− 16.6 ± 0.4	38.8 ± 4.4	N/A	N/A
PIC–Nal–IRI	158.8 ± 15.6	0.09 ± 0.03	− 14.8 ± 0.3	23.7 ± 2.2	48.0 ± 2.7	32.6 ± 2.6

*Nal* nanoliposome, *PIC–Nal* photoimmunoconjugate-nanoliposome, *Nal–IRI* nanoliposomal irinotecan, *PIC–Nal–IRI* photoimmunoconjugate-nanoliposomal irinotecan

^a^Encapsulation efficiency (%): The molar ratio of irinotecan within the liposome after purification to that added initially. ^b^Conjugation efficiency (%): The molar ratio of PIC conjugated onto the liposomal construct to that added initially

### Photoactivity of PIC–Nal and PIC–Nal–IRI

**Fig. 3:**
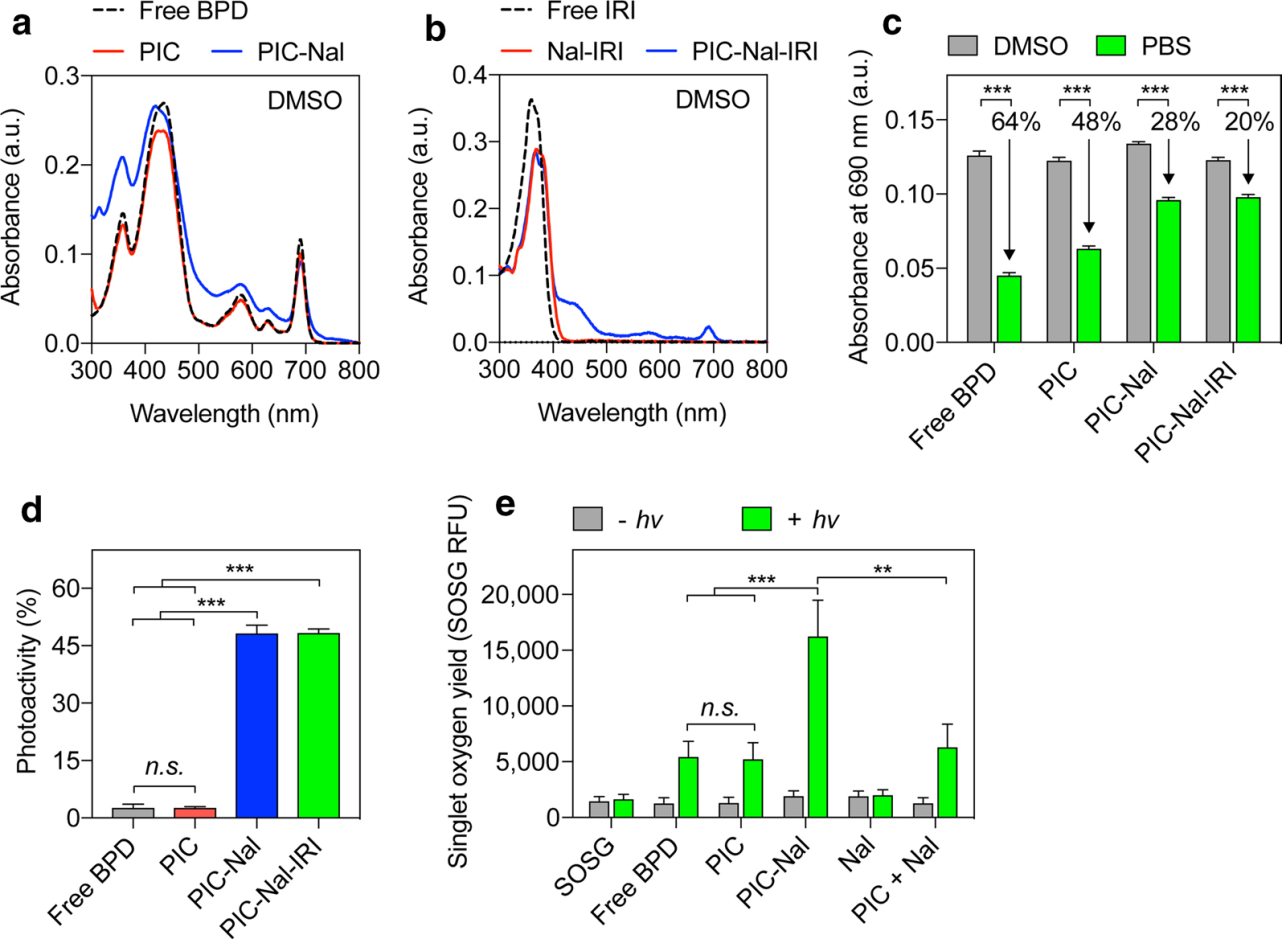
Photophysical and photochemical characterizations of PIC, PIC–Nal, and PIC–Nal–IRI. **a** Absorbance spectra of BPD, PIC, and PIC–Nal in DMSO showing overlapping main peaks centered at 435 nm (Soret band) and 690 nm (Q band; wavelength for light activation). **b** Absorbance spectra of irinotecan (IRI), Nal–IRI, and PIC–Nal–IRI in DMSO. **c** A comparison of the 690 nm absorbance value of BPD, PIC, PIC–Nal, and PIC–Nal–IRI in DMSO and PBS at a fixed BPD concentration. **d** Photoactivity of BPD, PIC, PIC–Nal, and PIC–Nal–IRI. Photoactivity is defined in the Methods section. **e** SOSG reports ^1^O_2_ production from free BPD, PIC, PIC–Nal, Nal, and ‘PIC + Nal’ in PBS with and without light activation at 690 nm. (*n* > 3; **P < 0.01, ***P < 0.001; *n.s.*: not significant; one-way ANOVA, Tukey’s posthoc test)

Hydrophobic BPD has a poor water solubility (< 0.05 mg/mL) and readily aggregates in biologically relevant media [32]. Conjugation of BPD to pegylated Cet enhances BPD solubility and allows precise control of BPD quenching and de-quenching [9]. We have previously shown that self-quenched BPD molecules on Cet can be de-quenched by cancer cells upon lysosomal proteolysis of the Cet, and thereby increasing the tumor specificity [9–11]. Prior to photoactivity evaluation, we confirmed that PIC, PIC–Nal and PIC–Nal–IRI do not alter the Q band of BPD (690 nm; Fig. 3a, b).

In Fig. 3c, due to the aggregation of BPD molecules in PBS, the absorbance values at 690 nm for free BPD and PIC in PBS were significantly reduced by ~ 64% and ~ 48%, respectively, compared to those fully dissolved in dimethyl sulfoxide. On the other hand, PIC–Nal showed a less pronounced (~ 28%) loss of absorbance value at 690 nm in PBS compared to that fully dissolved in DMSO (Fig. 3c). This is presumable due to the presence of PEG (~ 5 mol%) on the Nal that helps mitigate PIC aggregation in PBS. Loading of irinotecan into the aqueous core of PIC–Nal did not alter BPD’s absorbance value at 690 nm (Fig. 3c). Both free BPD and PIC showed poor photoactivity due to the static fluorescence quenching of BPD molecules as reported by us previously (Fig. 3d) [7, 9–11]. In contrast, PIC–Nal and PIC–Nal–IRI exhibit up to 45% of photoactivity. This suggests that BPD molecules on PIC–Nal and PIC–Nal–IRI are more readily activated by light for PIT in biologically relevant media compared to PIC (Fig. 3d). We next examined the singlet oxygen (^1^O_2_) yield of free BPD, PIC, and PIC–Nal using singlet oxygen sensor green (SOSG) probes. Upon light activation, the SOSG fluorescence intensity generated by PIC–Nal was significantly higher than that of free BPD, PIC and Nal (Fig. 3e), indicating that PIC–Nal has a higher ^1^O_2_ yield than BPD, PIC, or Nal. We also showed that simply mixing PIC with Nal (i.e., ‘PIC + Nal’) does not improve the ^1^O_2_ yield of PIC, confirming that the enhanced ^1^O_2_ yield of PIC-Nal relies on the successful click chemistry coupling of PICs onto Nal.

### Selectivity and uptake of PIC–Nal in cancer cells

**Fig. 4:**
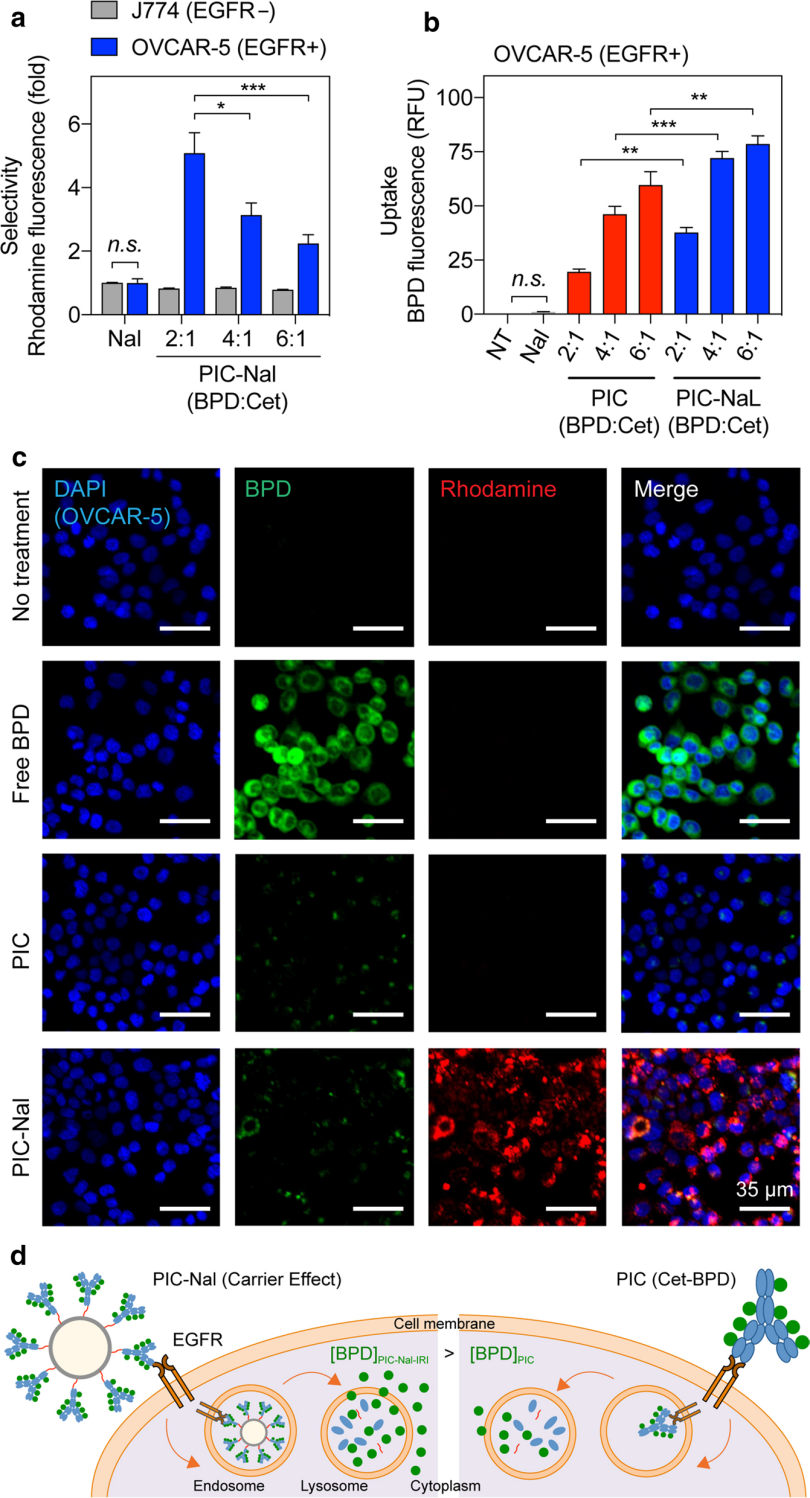
Selective binding, uptake, and imaging of PIC–Nal in cancer cells. **a** The selectivity of PIC–Nal was assessed in EGFR(−) J774 and EGFR(+) OVCAR-5 cells after 30 min of incubation. Nal alone was used as a control. The BPD:Cet ratio of PIC was varied (2:1, 4:1, 6:1). **b** The uptake of PIC–Nal and PIC in OVCAR-5 cells was assessed at 24 h after incubation, based on intracellular BPD fluorescence signal. **c** Representative fluorescence images of OVCAR-5 incubated with BPD, PIC, or PIC–Nal for 24 h. The BPD:Cet ratio of PIC was fixed at 6:1. Fluorescence signal of the nuclei (DAPI), BPD, and nanoliposome (rhodamine) shown in blue, green, and red, respectively (scale bar = 35 µm). **d** Depiction of the ‘carrier effect’ of PIC–Nal in EGFR(+) cancer cells. (*n* > 3; *P < 0.05, ***P < 0.001; *n.s.*: not significant; one-way ANOVA, Tukey’s posthoc test)

We next investigated if PIC–Nal can selectively deliver Nal to EGFR-overexpressing cells by comparing the selective uptake of PIC–Nal and Nal in EGFR(+) OVCAR-5 cells and EGFR( −) J774 macrophages at a fixed Nal concentration (based on rhodamine incorporation). After 30 min of incubation at 37** °**C, PIC–Nal uptake is 2- to 6-fold higher than Nal uptake in EGFR(+) OVCAR-5 cells (Fig. 4a). In contrast, PIC–Nal uptake was comparable to Nal uptake in EGFR(–) J774 macrophages. These results suggest PIC–Nal selectively binds to EGFR( +) cells over EGFR(–) cells. We also observed a reduction in the EGFR-targeting capability of PIC–Nal with increasing BPD:Cet ratio from 2:1 to 6:1 (Fig. 4a), indicating excessive loading of BPD on Cet can compromise the selectivity of the antibody. We next tested if cancer-selective PIC–Nal can improve the overall uptake of PIC in EGFR-overexpressing OVCAR-5 cells at 24 h post-incubation. Compared to PIC alone, we observed that PIC–Nal enhances (*P* < 0.05) the intracellular BPD uptake by 95%, 56%, and 32% at BPD:Cet molar ratios of 2:1, 4:1 and, 6:1, respectively (Fig. 4b). In contrast, this ‘carrier effect’ was not present in the low EGFR expressing U87 cells (Additional file 1: Figures S2, S3).

Leveraging the diagnostic capabilities of BPD fluorescence, we visualized the intracellular uptake of free BPD, PIC, and PIC–Nal in OVCAR-5 cells at 24 h post-incubation (Fig. 4c). Hydrophobic BPD can easily partition into the plasma membrane of both cancerous and non-malignant cells. Thus, it is not surprising that free BPD shows the highest uptake in OVCAR-5 cells compared to PIC and PIC–Nal. However, free BPD lacks selectivity against EGFR-overexpressing cancer cells*,* and thus will more likely induce off-target phototoxicity in vivo. Fluorescence microscopy images show that PIC–Nal modestly enhanced intracellular BPD accumulation compared to PIC alone (Fig. 4c), which agrees with our findings using the extraction method (Fig. 4b). Incubation with PIC–Nal led to a significant intracellular accumulation of Nal, indicated by the intense rhodamine fluorescence signals (Fig. 4c). This suggests the potential of delivering another therapeutic agent at a high payload using PIC–Nal. These studies verified that PIC–Nal not only enables EGFR-targeted delivery of Nal, but also serves as a platform to enhance PIC uptake in EGFR(+) cancer cells (Fig. 4d).

### PIC–Nal delivers irinotecan for synergistic photoimmuno-chemotherapy in vitro

**Fig. 5:**
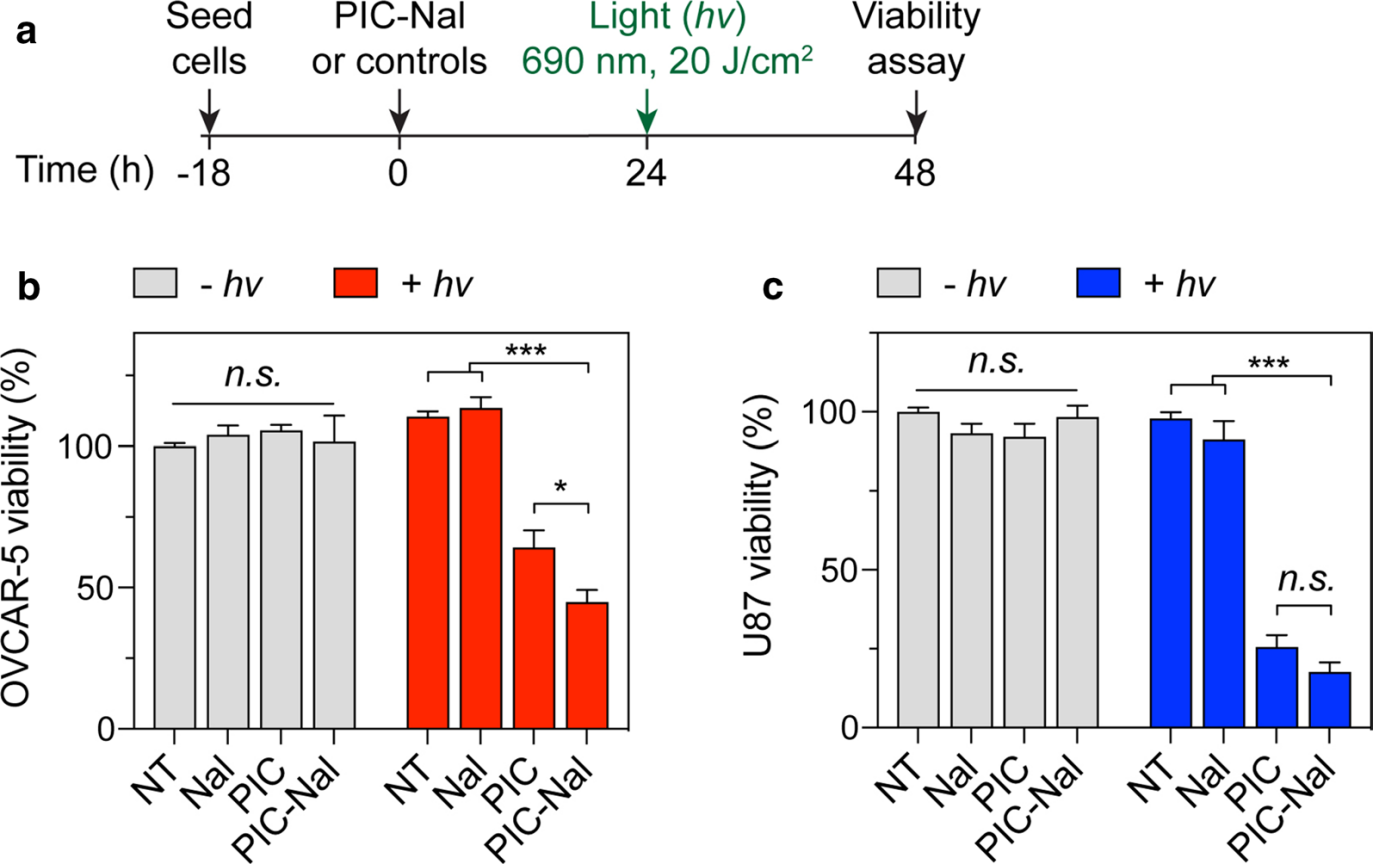
Phototoxicity of PIC–Nal and PIC in OVCAR-5 and U87 cells. **a** Cells were incubated with PIC or PIC–Nal at a fixed BPD concentration (0.25 µM) for 24 h prior to light activation (690 nm, 20 J/cm^2^, 150 mW/cm^2^). Cell viability was determined by MTT assay at 24 h post-light activation. PIC–Nal is more phototoxic than PIC in **b** high EGFR expressing OVCAR-5 but not in **c** low EGFR expressing U87. (*n* > 3; *P < 0.05, ***P < 0.001; *n.s.*: not significant; one-way ANOVA, Tukey’s posthoc test)

We investigated if PIC–Nal is more phototoxic than PIC using OVCAR-5 cells. U87 cells expressing lower EGFR levels served as a control (Additional file 1: Figure S1). At 24 h after light activation (20 J/cm^2^), PIC–Nal significantly reduced OVCAR-5 viability by ~ 60%, compared to ~ 35% viability reduction achieved by using PIC at a fixed BPD:Cet ratio of 6:1 (Fig. 5a, b). Similar results were observed using PIC and PIC–Nal with lower BPD:Cet ratios of 2:1 and 4:1 (Additional file 1: Figure S2). All samples, including PIC–Nal alone, PIC alone, and Nal alone, have negligible dark toxicity (Fig. 5b). In U87 cells, we observed no statistically significant difference in phototoxicity between PIC–Nal and PIC (Fig. 5c, Additional file 1: Figure S3), suggesting that the ‘carrier effect’ of PIC–Nal is, in part, dependent on the level of EGFR expression in cancer cells.

**Fig. 6:**
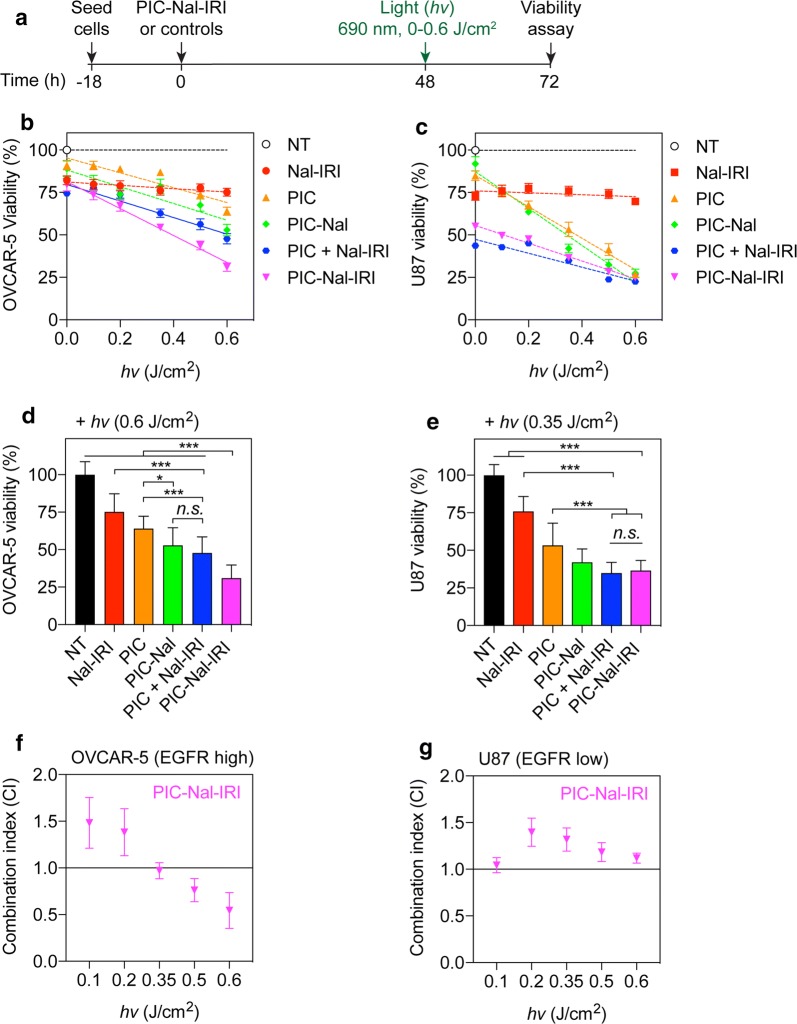
Combination of PIT and Nal–IRI in OVCAR-5 and U87 cells. **a** Cells were incubated with PIC–Nal–IRI or controls at a fixed BPD (1 µM) and irinotecan (7 µM) concentration for 48 h prior to light activation (690 nm, 10 mW/cm^2^, 0–0.6 J/cm^2^). **b** OVCAR-5 viability and **c** U87 viability were determined by MTT assay at 24 h post-light activation. The IC_50_ values of PIC–Nal are ~ 0.6 μM × J/cm^2^ and ~ 0.35 μM × J/cm^2^ for **d** OVCAR-5 and **e** U87 cells, respectively. **d**,** e** The reduction of cell viability was compared among the treatment groups. **f**,** g** Combination index (CI) was determined using CompuSyn software. The CI value quantitatively defines synergism (CI < 1), additive effect (CI = 1) and antagonism (CI > 1) effect of light-activated PIC–Nal–IRI in OVCAR-5 and U87 cells. (*n* > 3; *P < 0.05, ***P < 0.001; *n.s.*: not significant; one-way ANOVA, Tukey’s post hoc test)

PIC–Nal not only improved PIT efficacy against EGFR-overexpressing cancer cells, but provided us an opportunity to co-deliver irinotecan chemotherapy to further enhance treatment outcomes. We next evaluated the therapeutic efficacy of PIC–Nal–IRI at various light fluences (0–0.6 J/cm^2^) in OVCAR-5 and U87 cells (Fig. 6a). Control groups include Nal–IRI alone, PIC alone, PIC–Nal alone, and simply mixing PIC with Nal–IRI (‘PIC + Nal–IRI’) at fixed drug concentrations (i.e., irinotecan: 7 μM and BPD: 1 μM). The molar ratio of BPD-to-Cet was fixed at 6:1. In OVCAR-5 (Fig. 6b) and U87 cells (Fig. 6c), 72 h of Nal–IRI-treatment reduced cell viability by ~ 20–25%. Light activation of Nal–IRI alone did not alter the cell viability (*P* > 0.05). Both PIC and PIC–Nal alone showed minimal dark toxicity (< 15% viability reduction) (Fig. 6b, c). A light dose dependent reduction in cell viability was observed in both PIC- and PIC–Nal-treated cells. PIC–Nal was consistently found to be ~ 10–15% more phototoxic compared to PIC alone in OVCAR-5, but not in U87 cells. The IC_50_ of PIC–Nal upon light activation was approximately 0.6 μM × J/cm^2^ and 0.35 μM × J/cm^2^ for OVCAR-5 and U87 cells, respectively (Fig. 6b, c). In OVCAR-5 cells, while both PIC–Nal–IRI and ‘PIC +  Nal–IRI’ showed similar phototoxicity at 0.2 J/cm^2^ or below, we observed that PIC–Nal–IRI out-performs ‘PIC + Nal–IRI’ at or above 0.5 J/cm^2^ (Fig. 6b). At 0.6 μM × J/cm^2^, we showed that PIC–Nal–IRI is ~ 20% more cytotoxic than ‘PIC + Nal–IRI’ in OVCAR-5 cells (*P* < 0.001) (Fig. 6d). In contrary, both PIC–Nal–IRI and ‘PIC + Nal–IRI’ showed similar phototoxicity in U87 cells (Fig. 6e).

We further explored the combination interactions between the no-treatment (NT), PIC alone, Nal–IRI alone, and PIC–Nal–IRI groups (Fig. 6f, g). Using CompuSyn software and robust regression fits of the dose–response curve trend lines (R^2^ = 0.914–0.999) [33, 34], the combination index (CI) values were calculated to determine if combination of PIT and Nal–IRI using PIC–Nal–IRI is synergistic (*CI* < 1), additive (*CI* = 1), or antagonistic (*CI* > 1). In OVCAR-5 cells, combination of PIT and Nal–IRI using PIC–Nal–IRI is additive at 0.3 J/cm^2^ (*CI* 0.97 ± 0.09), and synergistic at 0.5 and 0.6 J/cm^2^ (*CI* 0.76 ± 0.12 and 0.54 ± 0.19, respectively). Therapeutic synergy was observed in a light dose dependent manner in OVCAR-5 cells (Fig. 6f), but not in U87 cells (*CI* 1.2 ± 0.1) (Fig. 6g).

### Multi-tier cellular targeting using PIC–Nal–IRI

**Fig. 7:**
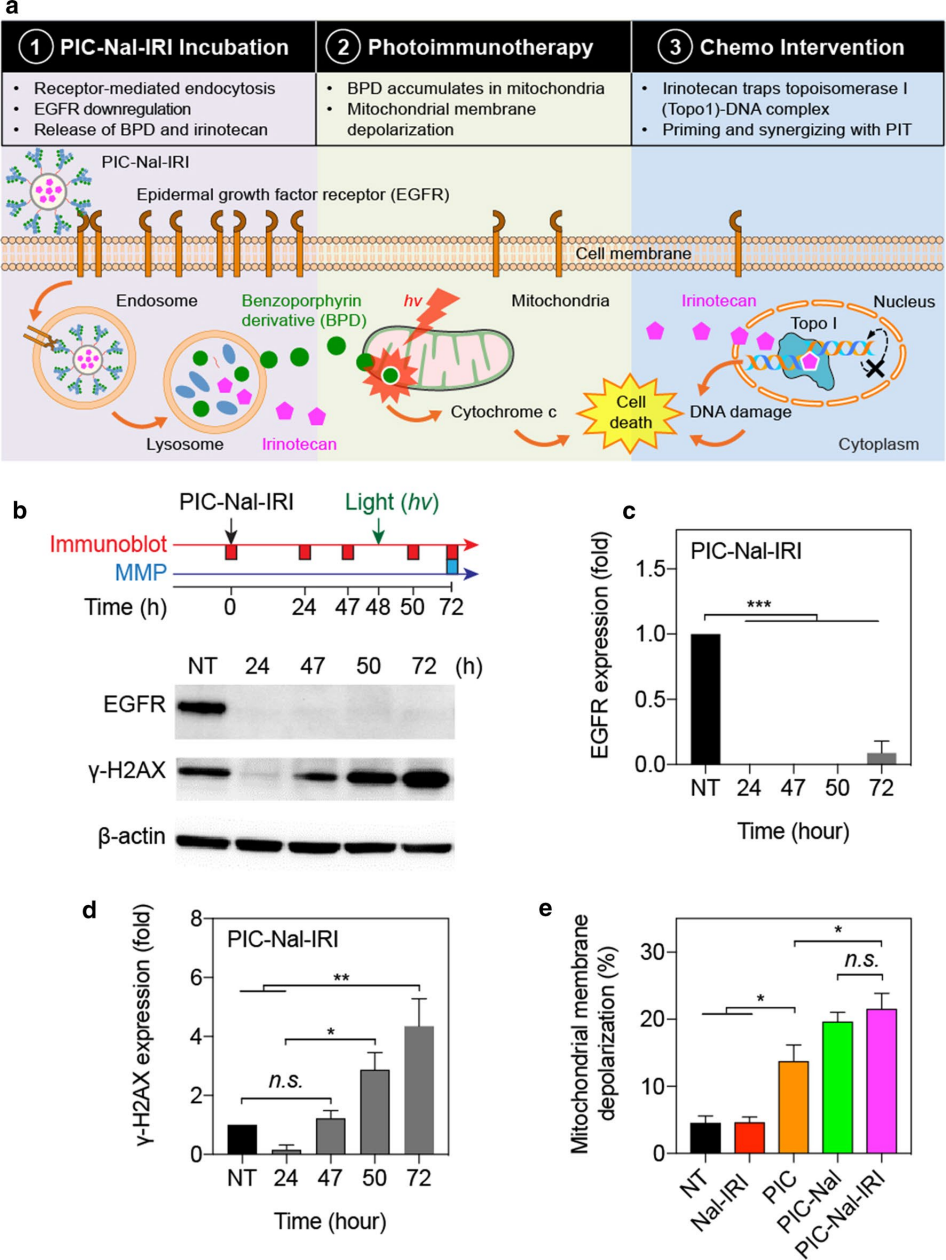
Multi-tier cancer targeting**.** **a** Schematic of multi-tier cancer targeting mechanism: (1) EGFR binding, endocytosis, and proteolysis of PIC–Nal–IRI, (2) PIT-mediated depolarization of mitochondrial membrane potential, and (3) irinotecan-induced DNA damage, leading to synergistic cell killing. **b** Immunoblotting of EGFR and γ-H2AX expression in OVCAR-5 cells at different time points after treatment. Quantitative analyses of normalized **c** EGFR and **d** γ-H2AX expressions in OVCAR-5 cells. **e** Mitochondrial membrane depolarization was assessed at 24 h post-light irradiation (0.35 J/cm^2^, 10 mW/cm^2^). (n = 3; *P < 0.05; **P < 0.01; ***P < 0.001; *n.s.*: not significant; one-way ANOVA, Tukey’s post hoc test)

The uniqueness of PIC–Nal–IRI lies, in part, in the multi-tier cellular targeting abilities. Three mechanistically distinct therapeutics (i.e., Cet, BPD, and irinotecan) were incorporated in PIC–Nal–IRI to target the EGFR, mitochondria, and DNA, respectively (Fig. 7a). Downregulation of total EGFR expression was observed after 24 h of PIC–Nal–IRI incubation and persisted throughout the treatment duration up to 72 h (Fig. 7b, c). Nal–IRI alone did not alter the EGFR expression (Additional file 1: Figure S4a). Irinotecan induced DNA damage was evaluated by monitoring the expression level of γ-H2AX [35]. PIC–Nal–IRI significantly upregulated γ-H2AX expression at 72 h post-incubation (Fig. 7d), indicating DNA double-strand breaks. γ-H2AX expression was found to be similar across all different groups (i.e., NT, Nal–IRI, PIC, and PIC–Nal–IRI) at 48 h post-incubation (Additional file 1: Figure S4b). We have recently shown that proteolyzed PIC co-localizes to mitochondria after 24 h and induces mitochondrial membrane potential (ΔΨm) depolarization upon light activation in glioma cells [9]. Here, we measured ΔΨm depolarization in OVCAR-5 cells at 24 h after light activation of PIC-Nal-IRI or controls (Fig. 7e). Light activation of PIC, PIC–Nal, or PIC–Nal–IRI all induced a high level of ΔΨm depolarization in OVCAR-5 cells (Fig. 7e). No ΔΨm depolarization was observed using Nal–IRI alone (Fig. 7e).

## Discussion

PIC is a promising and exciting tool in the armamentarium for cancer treatment, surgery, and imaging [1–3, 11]. However, the selectivity-uptake trade-off remains the major drawback limiting the application of PIC technology. Previous elegant works have shown that PIC (Cet-BPD) is highly selective against cancer cells overexpressing EGFR with 20-fold less accumulation in low EGFR cells [7, 36, 37]. The Cet-BPD also has a high tumor-to-normal tissue ratio (T/N) of 9.2, which mitigates bowel phototoxicity [11]. Despite high tumor selectivity, we recently discovered that the intracellular uptake of Cet-BPD is six fold less than that of free BPD in EGFR-overexpressing cancer cells, significantly reducing the anti-cancer phototoxicity by 20-fold [9]. To break through this selectivity-uptake trade-off, we introduced an engineering approach that leverages click chemistry to covalently tether large amounts of PICs (Cet-BPD) onto the surface of a Nal. We demonstrated that PIC–Nal is not only highly selective to EGFR-overexpressing OVCAR-5 cells with 2- to 5-fold less accumulation in macrophage cells, but also enhances PIC uptake in OVCAR-5 by ~ 20–30%, overcoming the selectivity-uptake trade-off and improving the overall PIT efficacy.

Similar results were observed by us previously using PIC-PLGA nanoparticles (PIC-NP) in OVCAR-5 and U87 cells, indicating the generalizability of this approach [10]. However, unlike PIC-NP, in this study, PIC–Nal did not enhance the PIC accumulation in low EGFR-expressing U87 cells. We speculate this discrepancy is attributed to the larger size (steric hindrance) and a lower PIC surface density of PIC–Nal (~ 150 nm, ~ 32 PICs per Nal), compared to the smaller size PIC-NP (~ 100 nm) with a higher PIC surface density (~ 75 PICs per NP). In fact, Gonzalez-Rodriguez et al. [38] have reported that cytoplasmic rigidity could limit the internalization of larger particles with radii above the optimal radius (typically around 50 nm) via receptor-mediated endocytosis. Vácha et al. [39] have also shown that increasing the antibody coverage on the surface of nanoparticles or the antibody-receptor binding affinity can improve receptor-mediated endocytosis. Based on these studies and our results, we believe that conjugation of PICs onto a nanoplatform to boost the cancer-selective PIC uptake is also contingent upon several important parameters, including particle size, PIC density, and PIC binding affinity of the nanoplatforms, which warrants further investigation and optimization.

PDT has been shown to reverse chemoresistance, synergize with chemotherapeutics and biologics, and overcome compensatory survival pathways used by cancer cells to evade treatment [40–44]. We have also shown that PDT synergizes with irinotecan to reduce metastatic burden and improve survival outcomes in pancreatic tumor mouse models via a two-way mechanism, in which (i) PDT photodamages ABCG2 drug efflux transporters to prevent irinotecan efflux, and (ii) irinotecan alleviates PDT-induced tumor hypoxia [21–23]. However, these studies utilized ‘non-targeted’ nanoliposomal irinotecan and ‘unquenched’ photosensitizers that are at a higher risk of normal tissue toxicity. A major advance of this study lies in our ability to reproducibly incorporate irinotecan into PIC–Nal for synergistic, targeted photoimmuno-chemotherpy. It has already been shown that the therapeutic synergy of combination treatments depends, in part, on the delivery of multiple drugs at a fixed molar ratio to cancer cells [45]. Here, we showed that PIC–Nal–IRI, co-delivering a fixed irinotecan-to-BPD molar ratio at 7:1, could be activated by light at low light fluences (0.5–0.6 J/cm^2^) for synergistic reduction of cancer cell viability (*CI* < 0.76). Further optimization of the irinotecan-to-PIC ratio in PIC–Nal–IRI is likely needed to maximize treatment outcomes in vivo. Another important finding is that PIC–Nal–IRI exhibits significantly higher OVCAR-5 cell phototoxicity by 20% (*P* < 0.001), compared to using the unconjugated mixtures of ‘PIC + Nal–IRI,’ which is an often-overlooked control during the development of multi-drug nanomedicine. In low EGFR expressing U87 cell, PIC–Nal–IRI and unconjugated mixtures of ‘PIC + Nal–IRI’ had similar phototoxicity at 0.35 J/cm^2^ (viability: 34.9 ± 2.0% vs. 36.5 ± 1.9%; *P* > 0.05), but both are superior to Nal–IRI alone or PIC-PIT alone (viability: 75.8 ± 2.8% vs. 53.5 ± 4.3%, respectively; *P* < 0.001). These observations suggest that, while combination of PIT and Nal–IRI is more effective in reducing cancer cell viability compared to their monotherapies, the co-packaging of PIC and irinotecan in a single nanoformulation might not be required in low EGFR-expressing tumors.

Combination treatments are most effective when targeting not only non-overlapping signaling pathways but also different subcellular components [28, 46]. Here, we integrated three mechanistically distinct, clinically used agents (Cet, BPD, and irinotecan) into a single nanoplatform to target the EGFR, mitochondria, and DNA, cooperatively. Similar to previous observations made by others and us using PIC or Cet alone [8, 9], we showed that PIC–Nal–IRI downregulates EGFR expression as soon as 24 h of administration. This also confirms that click conjugation of PIC onto Nal does not impair PIC’s ability to inhibit EGFR. It is well established that irinotecan-induced up-regulation of γ-H2AX, a prominent DNA damage marker, typically occurs at 48–72 h after incubation. Here, we showed that PIC–Nal–IRI elicits DNA breakage at 50 and 72 h after treatment. However, we observed that PIC–Nal–IRI transiently downregulates γ-H2AX expression in the first 24 h of incubation. This is presumably due to the activation of Cet-induced DNA repair pathways (e.g., Eme1) as shown by others [47]. Lastly, depolarization of the mitochondrial membrane was observed at 24 h after light activation of PIC–Nal–IRI, PIC-Nal, or PIC, but not with Nal–IRI alone, suggesting cytosolic mitochondrial photodamage is achieved primarily by PIC, as reported by us [9].

## Conclusion

In summary, the selectivity-uptake trade-off of PICs and the need of chemotherapy to enhance treatment outcomes are two major hurdles limiting the application of PIT for cancer management. This study introduces a light-activatable nanoplatform to overcome these challenges via a two-pronged approach. First, successful conjugation of PICs onto the surface of nanoliposomes overcomes the selectivity-uptake trade-off of PIC. Second, PIC–Nal–IRI offers a unique opportunity to target multiple major components of a cancer cell for synergistic therapeutic outcomes. Our in vitro results also point to valuable parameters (e.g., size, PIC density, and PIC binding affinity) to be optimized prior to advancing PIC–Nal–IRI to animal studies.

## Methods

### Photoimmunoconjugate (PIC) synthesis and characterization

Conjugation of BPD to Cet was achieved via carbodiimide chemistry [9, 10]. Briefly, Cet (152 kDa; 2 mg/mL) was pegylated with mPEG-NHS (40k; 16 mg/mL) overnight. Pegylated Cet was mixed with BPD *N*-hydroxysuccinimidyl ester (BPD-NHS) and azide-PEG4-*N*-hydroxysuccinimidyl ester (azide-PEG-NHS) at 1:3:2.5, 1:6:2.5, and 1:9:2.5 molar ratios for 20 h. The resulting PIC was purified using a 7 kDa MWCO Zeba™ spin desalting column that is pre-equilibrated with 30% DMSO, and concentrated with a 30 kDa centrifugal filter tube. The purity of PIC was confirmed to be over 99% using SDS-PAGE (Additional file 1: Figure S5). BPD concentration was determined by UV–Vis spectroscopy using established molar extinction coefficients (Additional file 1: Table S3). Antibody concentration was determined using BCA assay.

### Nanoliposome (Nal) synthesis and characterization

Nanoliposome (Nal) and nanoliposomal irinotecan (Nal–IRI) were prepared following freeze–thaw extrusion method [21, 22, 48, 49]. Briefly, cholesterol, dipalmitoylphosphatidylcholine (DPPC), distearoyl-phosphatidylethanolamine-methoxy polyethylene glycol (DSPE-mPEG2000), distearoyl-glycerophosphoethanolamine-N-dibenzocyclooctyl polyethylene glycol (DSPE-mPEG2000-DBCO), and dioleoylglycerophosphoglycerol (DOPG; Avanti) were mixed at a molar ratio of 2.8:6:0.4:0.2:0.6. For selectivity and uptake studies, 0.1 mol% of dipalmitoylglycero-phosphoethanolamine-N-(lissamine Rhodamine B sulfonyl) (16:0 Liss Rhod PE) was added to the lipid film. The dried lipid film was hydrated with deionized water with or without irinotecan (3 mM) prior to freeze–thaw cycling (4°C –45 °C). Multi-laminar nanoliposomes were then extruded through polycarbonate membrane (Whatman; 0.1 μm) at 45 °C and dialyzed against PBS to remove free irinotecan. Zetasizer NanoZS (Malvern) determined the size and zeta potential of Nals. The concentration of irinotecan was determined using UV–Vis spectroscopy and the established molar extinction coefficients (Additional file 1: Table S3) [21, 22, 48].

### Photoimmunoconjugate-nanoliposome (PIC-Nal) synthesis and characterization

Photoimmunoconjugate-nanoliposomes (PIC–Nal) and photoimmunoconjugate-nanoliposomal irinotecan (PIC–Nal–IRI) were synthesized via cooper-free click chemistry. Briefly, azide-containing PICs were mixed overnight with DBCO-containing Nal (or DBCO-containing Nal–IRI) at a molar ratio of 60:1. Sepharose CL-4B size exclusion chromatography was used to purify PIC–Nal and PIC–Nal–IRI. Drug concentrations were determined by UV–Vis spectroscopy and established molar extinction coefficients (Additional file 1: Table S3). Singlet oxygen sensor green (SOSG, 5 μM) was used to detect singlet oxygen (^1^O_2_) yield upon light irradiation of PIC–Nal–IRI or controls. BPD concentration is fixed at 5 μM. A microplate reader (BioTek) was used to acquire SOSG fluorescence signals (Ex/Em: 504/525 nm) before and after light irradiation (690 nm, 150 mW/cm^2^, 20 J/cm^2^). Photoactivity is defined as the maximal fluorescence intensity (FI) of photosensitizer in PBS divided by the maximal FI of photosensitizer in DMSO. The stability of the nanoformulations in PBS was determined by monitoring their hydrodynamic size and polydispersity index (PdI) over time. Irinotecan release from Nal–IRI and PIC–Nal–IRI was studied in 1% human serum at 37 °C under constant stirring using a dialysis setup described previously [21, 22].

### Selectivity, Uptake, and Phototoxicity of Photoimmunoconjugate-Nanoliposome (PIC–Nal)

Human ovarian cancer (OVCAR-5), human glioma (U87), and murine macrophage (J774) cell lines were purchased from ATCC and cultured in a 37 °C, 5% CO_2_ incubator with designated media. Cells were confirmed to be free of mycoplasma. For selectivity studies, EGFR(+) OVCAR-5 cells or EGFR(−) J774 cells were plated (400 k cells/35-mm Petri dish) and allowed to grow overnight. Cells were incubated with rhodamine-labeled PIC–Nal (or rhodamine-labeled Nal) at a fixed rhodamine concentration (0.5 μM) for 30 min (37 °C). After incubation, cells were washed twice with PBS and dissolved in Solvable™. The rhodamine fluorescence signals (Ex/Em: 545/610 nm) were acquired using a microplate reader to determine the selective binding of PIC–Nal. For uptake and phototoxicity studies, OVCAR-5 cells (200 k cells/35-mm dish) were incubated with PIC–Nal or controls (i.e., PIC alone, no-treatment) at a fixed BPD concentration (0.25 μM) for 24 h. For the uptake study, cells were washed twice with PBS and dissolved in Solvable™. The BPD fluorescence signals (Ex/Em: 435/690 nm) were acquired using a microplate reader to quantify the uptake of PIC–Nal. In another set of experiment, washed cells were fixed with 4% paraformaldehyde, and stained with DAPI. Cells were imaged with the LionHeart Imager (BioTek) using the 10 x objective to visualize the BPD signal (Ex/Em: 422/690 nm) and the DAPI signal (Ex/Em: 358/461 nm). BPD fluorescence intensity was quantified using ImageJ [50]. For phototoxicity studies, cells were irradiated with a 690 nm laser (20 J/cm^2^, 150 mW/cm^2^) at 24 h post-incubation of PIC–Nal or controls. Cell viability was determined by MTT (3-(4,5-dimethylthiazol-2-yl)-2,5-diphenyltetrazolium bromide) assay (Thermo) at 24 h post-light activation.

### Photoimmuno-chemotherapy efficacy

To assess photoimmuno-chemotherapy efficacy, OVCAR-5 (5 k cells/well) and U87 cells (7 k cells/well), cultured in black-wall flat bottom 96-well plates, were incubated with PIC–Nal–IRI or controls at fixed drug concentrations (i.e., 1 μM of BPD and 7 μM of irinotecan) for 48 h prior to light activation (690 nm, 0–0.6 J/cm^2^, 10 mW/cm^2^; Modulight). Cell viability was determined by MTT assay at 24 h post-light activation. Mitochondrial membrane potential (ΔΨm) was examined via TMRE assay (Abcam). For western blot analyses, cell lysates (20 µg) were separated on 4–12% precast Bis–Tris protein gels and transferred onto a PVDF membrane. Subsequent to blocking with 5% BSA or milk in TBST solution, proteins were further detected using antibodies against EGFR (1:1000, Cell Signaling #2239) and γ-H2AX (1:500, EMP #05636). Anti β-actin antibodies (1:5000, Cell Signaling #3700) were used for the loading control. Visualization of protein bands was developed via chemiluminescence (SuperSignal) with exposure to a Gel Imager (ProteinSimple).

### Statistical analysis

All experiments were carried out at least in triplicates. Specific tests and number of repeats are indicated in the figure captions. Results were shown with mean ± standard error of the mean (SEM). Statistical analyses were performed using GraphPad Prism (GraphPad Software).

## Supplementary information

**Additional file 1: Table S1.** Synthesis of photoimmunoconjugates with different BPD-to-Cetuximab (BPD:Cet) ratios. **Table S2.** Physical characterization of nanoliposome (Nal) and photoimmunoconjugate-nanoliposome (PIC-Nal) with varying BPD-to-Cetuximab (BPD:Cet) ratios of PIC. **Table S3.** Molar extinction coefficients (*ε*) and equations used to determine the irinotecan concentration (*C*_IRI_) and BPD concentration (*C*_BPD_) of PIC-Nal-IRI in DMSO using Beer-Lambert law. **Figure S1.** Immunoblotting of EGFR in human OVCAR-5 and U87 cells. Whole cell extracts (20 µg) were loaded into each lane. β-actin was used as loading control. OVCAR-5 cell line has a higher EGFR expression compared to U87 cells. **Figure S2.** Phototoxicity of photoimmunoconjugate-nanoliposome (PIC-Nal) at different BPD:Cet ratios in human ovarian cancer cells (OVCAR-5). Cells were incubated with PIC or PIC-Nal at a fixed BPD concentration of 0.25 µM for 24 h before light activation at 690 nm (20 J/cm^2^, 150 mW/cm^2^, bottom illumination). Cell viability was determined by MTT assay at 24 h after photoimmunotherapy (PIT). (*n* > 3; *P < 0.05; one-way ANOVA, Tukey’s posthoc test). **Figure S3.** Intracellular BPD fluorescence signals of PIC and PIC-Nal (at different BPD:Cet ratios) were evaluated in human glioma cells (U87) via extraction method. Cells were incubated with PIC or PIC-Nal at a fixed BPD concentration of 0.25 µM for 24 h prior to extraction (*n* > 3; *n.s.*: not significant; one-way ANOVA, Tukey’s posthoc test). **Figure S4.** Immunoblotting of EGFR and γ-H2AX expressions in OVCAR-5 cells at 24 h and 47 h after incubation of PIC, Nal-IRI, and PIC-Nal-IRI. Whole cell extracts (20 µg) were loaded into each lane. β-actin was used as a loading control. (a) Downregulation of EGFR was most pronounced when treated with PIC-Nal-IRI. (b) The γ-H2AX expression remained at a similar level across different treatment groups. (*n *= 3; *P < 0.05; **P < 0.01; ***P < 0.001; one-way ANOVA, Tukey’s posthoc test). **Figure S5. **The purity of Cet-BPD was assessed by gel fluorescence imaging analysis following sodium dodecyl sulfate polyacrylamide gel electrophoresis (SDS-PAGE). (a) Coomassie blue staining of SDS-PAGE for visualization of the standards (Ladder), Cet, Cet-BPD (PIC), and BPD. (b) Gel fluorescence imaging (Em: 690 nm) of SDS-PAGE shows < 1% free BPD impurity in PIC; fluorescence intensity was quantified using ImageJ.

## Data Availability

The datasets used and/or analyzed during the current study are available from the corresponding author on reasonable request.

## References

[1] van Dongen GA, Visser GW, Vrouenraets MB (2004). Photosensitizer-antibody conjugates for detection and therapy of cancer. Adv Drug Deliv Rev..

[2] Mitsunaga M, Ogawa M, Kosaka N, Rosenblum LT, Choyke PL, Kobayashi H (2011). Cancer cell-selective in vivo near infrared photoimmunotherapy targeting specific membrane molecules. Nat Med..

[3] Schmidt S, Wagner U, Oehr P, Krebs D (1992). Clinical use of photodynamic therapy in gynecologic tumor patients–antibody-targeted photodynamic laser therapy as a new oncologic treatment procedure. Zentralbl Gynakol..

[4] Mew D, Wat CK, Towers GH, Levy JG (1983). Photoimmunotherapy: treatment of animal tumors with tumor-specific monoclonal antibody-hematoporphyrin conjugates. J Immunol..

[5] van Dam GM, Themelis G, Crane LM, Harlaar NJ, Pleijhuis RG, Kelder W, Sarantopoulos A, de Jong JS, Arts HJ, van der Zee AG (2011). Intraoperative tumor-specific fluorescence imaging in ovarian cancer by folate receptor-alpha targeting: first in-human results. Nat Med..

[6] Normanno N, De Luca A, Bianco C, Strizzi L, Mancino M, Maiello MR, Carotenuto A, De Feo G, Caponigro F, Salomon DS (2006). Epidermal growth factor receptor (EGFR) signaling in cancer. Gene.

[7] Savellano MD, Hasan T (2003). Targeting cells that overexpress the epidermal growth factor receptor with polyethylene glycolated BPD verteporfin photosensitizer immunoconjugates. Photochem Photobiol..

[8] Abu-Yousif AO, Moor ACE, Zheng X, Savellano MD, Yu W, Selbo PK, Hasan T (2012). Epidermal growth factor receptor-targeted photosensitizer selectively inhibits EGFR signaling and induces targeted phototoxicity in ovarian cancer cells. Cancer Lett.

[9] Inglut CT, Baglo Y, Liang BJ, Cheema Y, Stabile J, Woodworth GF, Huang H-C (2019). Systematic evaluation of light-activatable biohybrids for anti-glioma photodynamic therapy. J Clin Med..

[10] Huang HC, Pigula M, Fang Y, Hasan T (2018). Immobilization of photo-immunoconjugates on nanoparticles leads to enhanced light-activated biological effects. Small..

[11] Spring BQ, Abu-Yousif AO, Palanisami A, Rizvi I, Zheng X, Mai Z, Anbil S, Sears RB, Mensah LB, Goldschmidt R (2014). Selective treatment and monitoring of disseminated cancer micrometastases in vivo using dual-function, activatable immunoconjugates. Proc Natl Acad Sci.

[12] Kessel D, Castelli M (2001). Evidence that bcl-2 is the target of three photosensitizers that induce a rapid apoptotic response. Photochem Photobiol..

[13] Kessel D, Luo Y (1998). Mitochondrial photodamage and PDT-induced apoptosis. J Photochem Photobiol, B.

[14] Kessel D, Luo Y, Deng Y, Chang CK (1997). The role of subcellular localization in initiation of apoptosis by photodynamic therapy. Photochem Photobiol.

[15] Peer D, Karp JM, Hong S, Farokhzad OC, Margalit R, Langer R (2007). Nanocarriers as an emerging platform for cancer therapy. Nat Nanotechnol.

[16] Vredenburgh JJ, Desjardins A, Reardon DA, Friedman HS (2009). Experience with irinotecan for the treatment of malignant glioma. Neuro-Oncology..

[17] Pommier Y (2006). Topoisomerase I inhibitors: camptothecins and beyond. Nat Rev Cancer..

[18] Herrlinger U, Schafer N, Steinbach JP, Weyerbrock A, Hau P, Goldbrunner R, Friedrich F, Rohde V, Ringel F, Schlegel U (2016). Bevacizumab plus irinotecan versus temozolomide in newly diagnosed O6-methylguanine-DNA methyltransferase nonmethylated glioblastoma: the randomized GLARIUS trial. J Clin Oncol..

[19] Saif MW (2014). MM-398 achieves primary endpoint of overall survival in phase III study in patients with gemcitabine refractory metastatic pancreatic cancer. Jop.

[20] Parchment RE, Pessina A (1998). Topoisomerase I inhibitors and drug resistance. Cytotechnology.

[21] Huang HC, Rizvi I, Liu J, Anbil S, Kalra A, Lee H, Baglo Y, Paz N, Hayden D, Pereira S (2018). Photodynamic priming mitigates chemotherapeutic selection pressures and improves drug delivery. Cancer Res..

[22] Huang HC, Mallidi S, Liu J, Chiang CT, Mai Z, Goldschmidt R, Ebrahim-Zadeh N, Rizvi I, Hasan T (2016). Photodynamic therapy synergizes with irinotecan to overcome compensatory mechanisms and improve treatment outcomes in pancreatic cancer. Cancer Res..

[23] Pigula M, Huang HC, Mallidi S, Anbil S, Liu J, Mai Z, Hasan T (2019). Size-dependent tumor response to photodynamic therapy and irinotecan monotherapies revealed by longitudinal ultrasound monitoring in an orthotopic pancreatic cancer model. Photochem Photobiol..

[24] Carter KA, Luo D, Razi A, Geng J, Shao S, Ortega J, Lovell JF (2016). Sphingomyelin liposomes containing Porphyrin-phospholipid for irinotecan chemophototherapy. Theranostics..

[25] Chabner BA, Roberts TG (2005). Timeline: chemotherapy and the war on cancer. Nat Rev Cancer..

[26] Lopez JS, Banerji U (2017). Combine and conquer: challenges for targeted therapy combinations in early phase trials. Nat Rev Clin Oncol..

[27] Sakhrani NM, Padh H (2013). Organelle targeting: third level of drug targeting. Drug Design Dev Ther..

[28] Barua S, Mitragotri S (2013). Synergistic targeting of cell membrane, cytoplasm, and nucleus of cancer cells using rod-shaped nanoparticles. ACS Nano.

[29] Huang HC, Hasan T (2014). The nano world in photodynamic therapy austin. J Nanomed Nanotechnol..

[30] Wonder E, Simón-Gracia L, Scodeller P, Majzoub RN, Kotamraju VR, Ewert KK, Teesalu T, Safinya CR (2018). Competition of charge-mediated and specific binding by peptide-tagged cationic liposome-DNA nanoparticles in vitro and in vivo. Biomaterials.

[31] Miller CR, Bondurant B, McLean SD, McGovern KA, O'Brien DF (1998). Liposome-cell interactions in vitro: effect of liposome surface charge on the binding and endocytosis of conventional and sterically stabilized liposomes. Biochemistry.

[32] Chen B, Pogue BW, Hasan T (2005). Liposomal delivery of photosensitising agents. Expert Opin Drug Deliv..

[33] Chou TC (2006). Theoretical basis, experimental design, and computerized simulation of synergism and antagonism in drug combination studies. Pharmacol Rev..

[34] Chou TC, Talalay P (1984). Quantitative analysis of dose-effect relationships: the combined effects of multiple drugs or enzyme inhibitors. Adv Enzyme Regul..

[35] Kuo LJ, Yang LX (2008). Gamma-H2AX - a novel biomarker for DNA double-strand breaks. Vivo..

[36] Abu-Yousif AO, Moor AC, Zheng X, Savellano MD, Yu W, Selbo PK, Hasan T (2012). Epidermal growth factor receptor-targeted photosensitizer selectively inhibits EGFR signaling and induces targeted phototoxicity in ovarian cancer cells. Cancer Lett..

[37] Savellano MD, Hasan T (2005). Photochemical targeting of epidermal growth factor receptor: a mechanistic study. Clin Cancer Res..

[38] Gonzalez-Rodriguez D, Barakat AI (2015). Dynamics of receptor-mediated nanoparticle internalization into endothelial cells. PLoS ONE.

[39] Vácha R, Martinez-Veracoechea FJ, Frenkel D (2011). Receptor-mediated endocytosis of nanoparticles of various shapes. Nano Lett..

[40] Spring BQ, Rizvi I, Xu N, Hasan T (2015). The role of photodynamic therapy in overcoming cancer drug resistance. Photochem Photobiol Sci..

[41] Baglo Y, Liang BJ, Robey RW, Ambudkar SV, Gottesman MM, Huang H-C (2019). Porphyrin-lipid assemblies and nanovesicles overcome ABC transporter-mediated photodynamic therapy resistance in cancer cells. Cancer Lett..

[42] Gallagher-Colombo SM, Miller J, Cengel KA, Putt ME, Vinogradov SA, Busch TM (2015). Erlotinib pretreatment improves photodynamic therapy of non-small cell lung carcinoma xenografts via multiple mechanisms. Cancer Res..

[43] Luo D, Goel S, Liu H-J, Carter KA, Jiang D, Geng J, Kutyreff CJ, Engle JW, Huang W-C, Shao S (2017). Intrabilayer 64cu labeling of photoactivatable, doxorubicin-loaded stealth liposomes. ACS Nano.

[44] Rizvi I, Celli JP, Evans CL, Abu-Yousif AO, Muzikansky A, Pogue BW, Finklestein D, Hasan T (2010). Synergistic enhancement of carboplatin efficacy with photodynamic therapy in a three-dimensional model for micrometastatic ovarian cancer. Cancer Res..

[45] Tolcher AW, Mayer LD (2018). Improving combination cancer therapy: the CombiPlex((R)) development platform. Future Oncol..

[46] Fernald K, Kurokawa M (2013). Evading apoptosis in cancer. Trends Cell Biol.

[47] Weinandy A, Piroth MD, Goswami A, Nolte K, Sellhaus B, Gerardo-Nava J, Eble M, Weinandy S, Cornelissen C, Clusmann H (2014). Cetuximab induces eme1-mediated DNA repair: a novel mechanism for cetuximab resistance. Neoplasia..

[48] Huang HC, Liu J, Baglo Y, Rizvi I, Anbil S, Pigula M, Hasan T (2018). Mechanism-informed repurposing of minocycline overcomes resistance to topoisomerase inhibition for peritoneal carcinomatosis. Mol Cancer Ther..

[49] Inglut CT, Gaitan B, Najafali D, Abad Lopez I, Connolly NP, Orsila S, Perttilä R, Woodworth GF, Chen Y, Huang H-C (2019). Predictors and limitations of the penetration depth of photodynamic effects in the rodent brain. Photochem Photobiol.

[50] Schneider CA, Rasband WS, Eliceiri KW (2012). NIH Image to ImageJ: 25 years of image analysis. Nat Methods..

