# Supplementary material for: Breaking the selectivity-uptake trade-off of photoimmunoconjugates with nanoliposomal irinotecan for synergistic multi-tier cancer targeting
Source: J Nanobiotechnology. 2020 Jan 2;18:1. doi: 10.1186/s12951-019-0560-5 (PMC6939330; doi:10.1186/s12951-019-0560-5)
Supplement: Supplementary file 1 — Additional file 1: Table S1. Synthesis of photoimmunoconjugates with different BPD-to-Cetuximab (BPD:Cet) ratios. Table S2. Physical characterization of nanoliposome (Nal) and photoimmunoconjugate-nanoliposome (PIC-Nal) with varying BPD-to-Cetuximab (BPD:Cet) ratios of PIC. Table S3. Molar extinction coefficients (ε) and equations used to determine the irinotecan concentration (CIRI) and BPD concentration (CBPD) of PIC-Nal-IRI in DMSO using Beer-Lambert law. Figure S1. Immunoblotting of EGFR in human OVCAR-5 and U87 cells. Whole cell extracts (20 µg) were loaded into each lane. β-actin was used as loading control. OVCAR-5 cell line has a higher EGFR expression compared to U87 cells. Figure S2. Phototoxicity of photoimmunoconjugate-nanoliposome (PIC-Nal) at different BPD:Cet ratios in human ovarian cancer cells (OVCAR-5). Cells were incubated with PIC or PIC-Nal at a fixed BPD concentration of 0.25 µM for 24 h before light activation at 690 nm (20 J/cm2, 150 mW/cm2, bottom illumination). Cell viability was determined by MTT assay at 24 h after photoimmunotherapy (PIT). (n > 3; *P < 0.05; one-way ANOVA, Tukey’s posthoc test). Figure S3. Intracellular BPD fluorescence signals of PIC and PIC-Nal (at different BPD:Cet ratios) were evaluated in human glioma cells (U87) via extraction method. Cells were incubated with PIC or PIC-Nal at a fixed BPD concentration of 0.25 µM for 24 h prior to extraction (n > 3; n.s.: not significant; one-way ANOVA, Tukey’s posthoc test). Figure S4. Immunoblotting of EGFR and γ-H2AX expressions in OVCAR-5 cells at 24 h and 47 h after incubation of PIC, Nal-IRI, and PIC-Nal-IRI. Whole cell extracts (20 µg) were loaded into each lane. β-actin was used as a loading control. (a) Downregulation of EGFR was most pronounced when treated with PIC-Nal-IRI. (b) The γ-H2AX expression remained at a similar level across different treatment groups. (n = 3; *P < 0.05; **P < 0.01; ***P < 0.001; one-way ANOVA, Tukey’s posthoc test). Figure S5. The purity of Ce [file 12951_2019_560_MOESM1_ESM.docx]

**Additional file 1**

**Table S1**

| **Table S1.** Synthesis of photoimmunoconjugates with different BPD-to-Cetuximab (BPD:Cet) ratios | | |
| --- | --- | --- |
| Reaction stoichiometry (BPD:Cet) | Final Molar Ratio (BPD:Cet) | Conjugation Efficiency (%)* |
| 9:1 | 6.13 ± 0.43 | 68.1 ± 1.54% |
| 6:1 | 3.87 ± 0.35 | 64.5 ± 1.01% |
| 3:1 | 2.04 ± 0.24 | 68.0 ± 0.96% |
| *Conjugation Efficiency (%): The molar ratio of BPD conjugated onto Cet to that added initially. | | |

**Table S2**

| **Table S2.** Physical characterization of nanoliposome (Nal) and photoimmunoconjugate-nanoliposome (PIC-Nal) with varying BPD-to-Cetuximab (BPD:Cet) ratios of PIC. | | | | | |
| --- | --- | --- | --- | --- | --- |
| Formulation  (BPD:Cet) | Size (d.nm) | PdI | Zeta potential (mV) | Conjugation Efficiency (%)* | Number of PIC *per* Nal |
| Nal | 126.5 ± 3.5 | 0.08 ± 0.01 | -19.6 ± 0.9 | N/A | N/A |
| PIC-Nal (6:1) | 142.5 ± 5.9 | 0.06 ± 0.01 | -13.6 ± 0.6 | 66.6 ± 2.3 | 39.9 ± 1.4 |
| PIC-Nal (4:1) | 139.8 ± 6.3 | 0.08 ± 0.01 | -13.4 ± 0.1 | 65.8 ± 4.3 | 39.9 ± 3.5 |
| PIC-Nal (2:1) | 141.5 ± 6.8 | 0.06 ± 0.02 | -13.7 ± 0.1 | 71.0 ± 2.7 | 42.6 ± 2.6 |
| *Conjugation Efficiency (%): The molar ratio of PIC conjugated onto Nal to that added initially. | | | | | |

**Table S3**

| **Table S3**. Molar extinction coefficients (*ε*) and equations used to determine the irinotecan concentration (*C*_IRI_) and BPD concentration (*C*_BPD_) of PIC-Nal-IRI in DMSO using Beer-Lambert law. | | | | |
| --- | --- | --- | --- | --- |
| Absorbance (*Abs.*, nm) | 367 nm | 348 nm | 435 nm | 687 nm |
| Irinotecan, *ε*_IRI_ (M^-1^ cm^-1^) | 21,484 | 24,473 | 0 | 0 |
| BPD, *ε*_BPD_ (M^-1^ cm^-1^) | 35,759 | 36,726 | 80,500 | 34,895 |
| $C_{IRI}= \left[ \frac{\left( {Abs}_{367}-{Abs}_{435} \right)}{\varepsilon_{IRI @ 367}\times l}\times\frac{\varepsilon_{BPD @ 367}}{\varepsilon_{BPD@ 435}}+ \frac{\left( {Abs}_{384}-{Abs}_{435} \right)}{\varepsilon_{IRI @ 384}\times l}\times\frac{\varepsilon_{BPD @ 384}}{\varepsilon_{BPD@ 435}} \right]\times\frac{1}{2}$  $C_{BPD}=\left[ \frac{{Abs}_{435}}{\varepsilon_{BPD @ 435}\times l}+\frac{{Abs}_{687}}{\varepsilon_{BPD @ 687}\times l} \right]\times\frac{1}{2}$ | | | | |

**Figure S1**


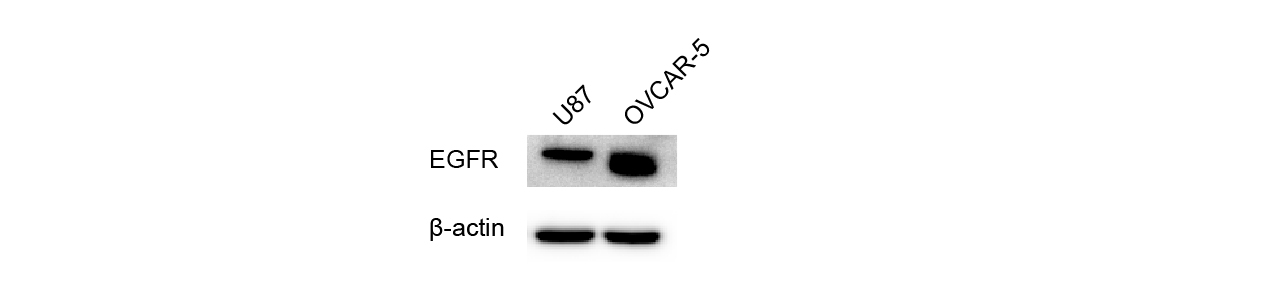


**Figure S1.** **Immunoblotting of EGFR in human OVCAR-5 and U87 cells.** Whole cell extracts (20 µg) were loaded into each lane. β-actin was used as loading control. OVCAR-5 cell line has a higher EGFR expression compared to U87 cells.

**Figure S2**

**
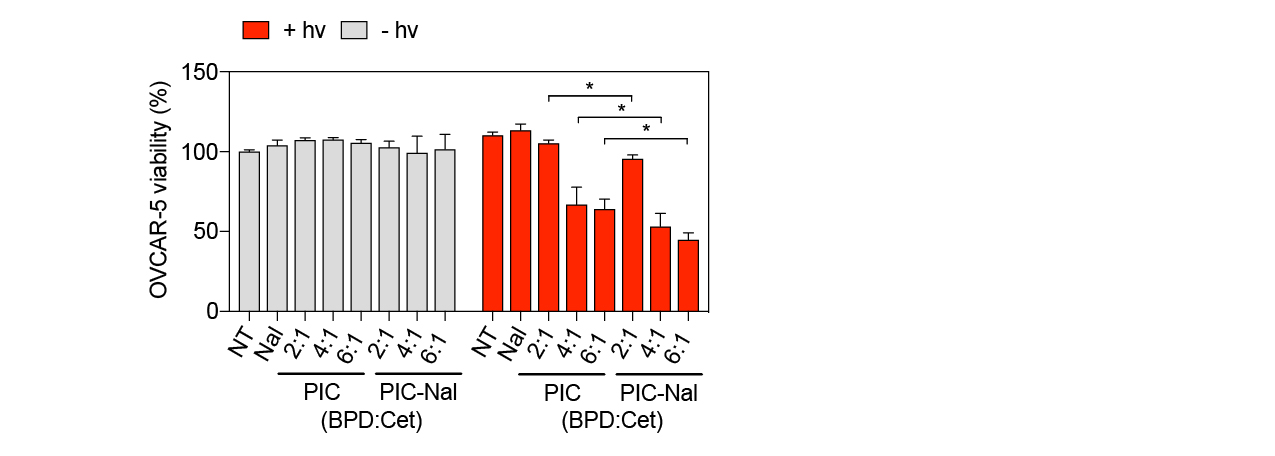
**

**Figure S2.** **Phototoxicity of photoimmunoconjugate-nanoliposome (PIC-Nal) at different BPD:Cet ratios in human ovarian cancer cells (OVCAR-5).** Cells were incubated with PIC or PIC-Nal at a fixed BPD concentration of 0.25 µM for 24 hours before light activation at 690 nm (20J/cm^2^, 150 mW/cm^2^, bottom illumination). Cell viability was determined by MTT assay at 24 hours after photoimmunotherapy (PIT). (*n*>3; **P*<0.05; one-way ANOVA, Tukey’s posthoc test)

**Figure S3**


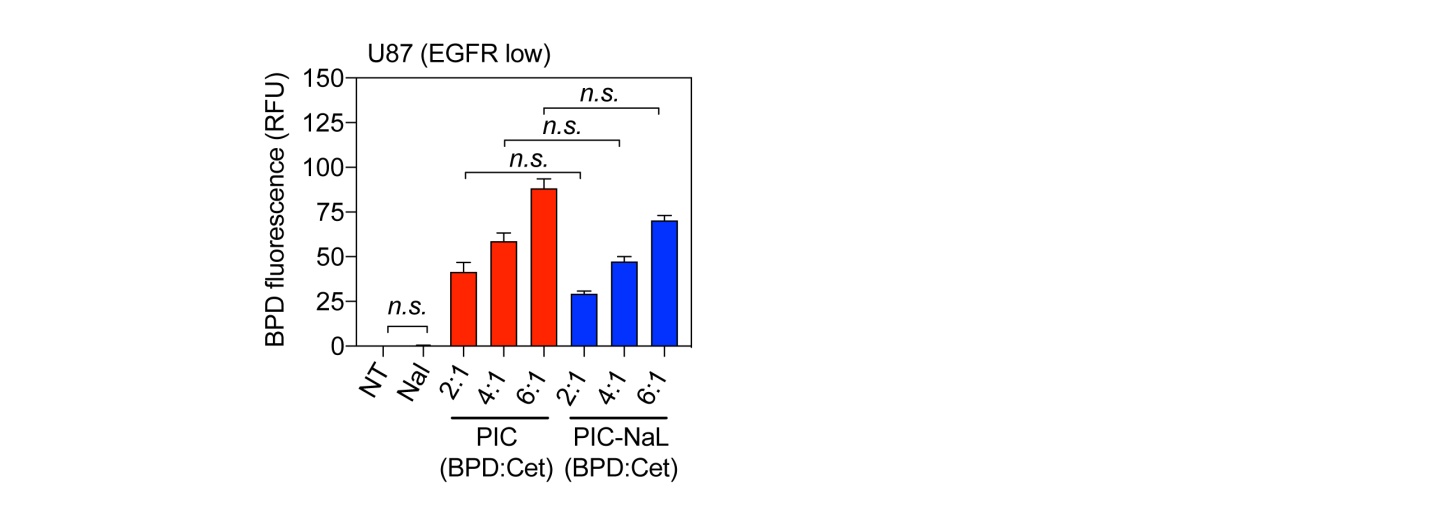


**Figure S3.** **Intracellular BPD fluorescence signals of PIC and PIC-Nal at different BPD:Cet ratios were evaluated in human glioma cells (U87) *via* extraction method.** Cells were incubated with PIC or PIC-Nal at a fixed BPD concentration of 0.25 µM for 24 hours prior to extraction (*n*>3; *n.s.*: not significant; one-way ANOVA,Tukey’s posthoc test).

**Figure S4**


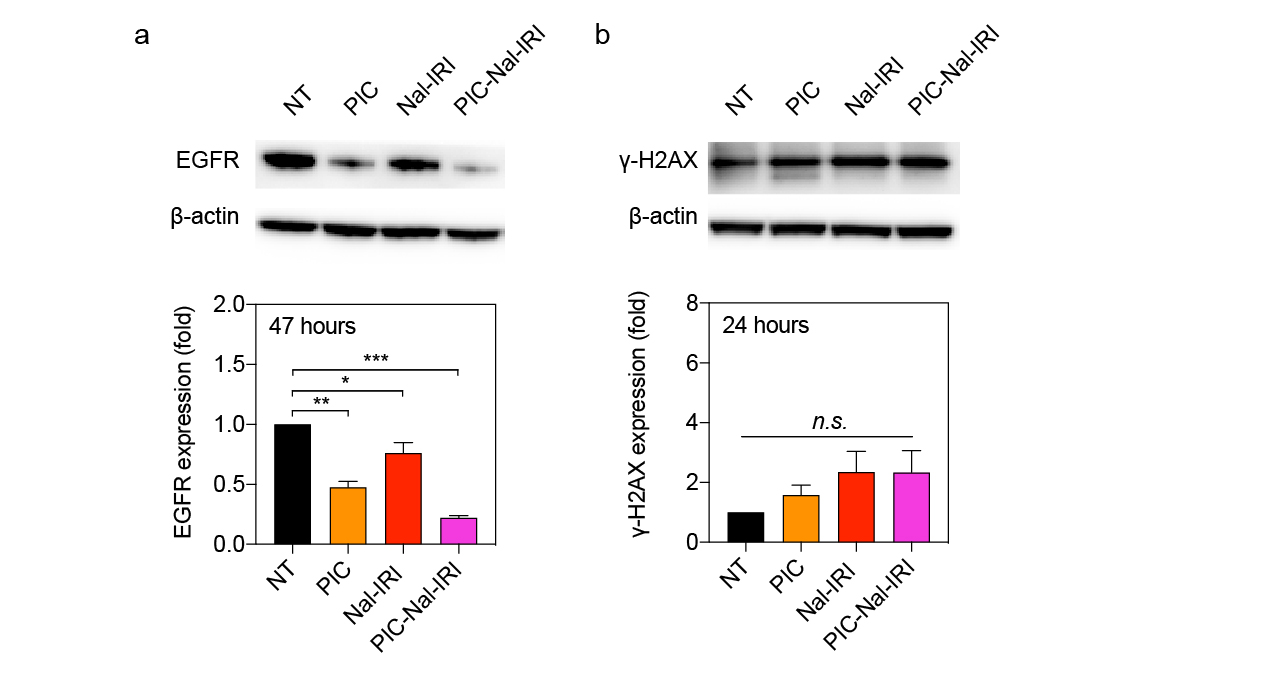


**Figure S4. Immunoblotting of EGFR and γ-H2AX expressions in OVCAR-5 cells at 24 hours and 47 hours after incubation of PIC, Nal-IRI, and PIC-Nal-IRI.** Whole cell extracts (20 µg) were loaded into each lane. β-actin was used as a loading control. (**a**) Downregulation of EGFR was most pronounced when treated with PIC-Nal-IRI. (**b**) The γ-H2AX expression remained at a similar level across different treatment groups. (*n*=3; **P*<0.05; ***P*<0.01; ****P*<0.001; one-way ANOVA, Tukey’s posthoc test).

**Figure S5**


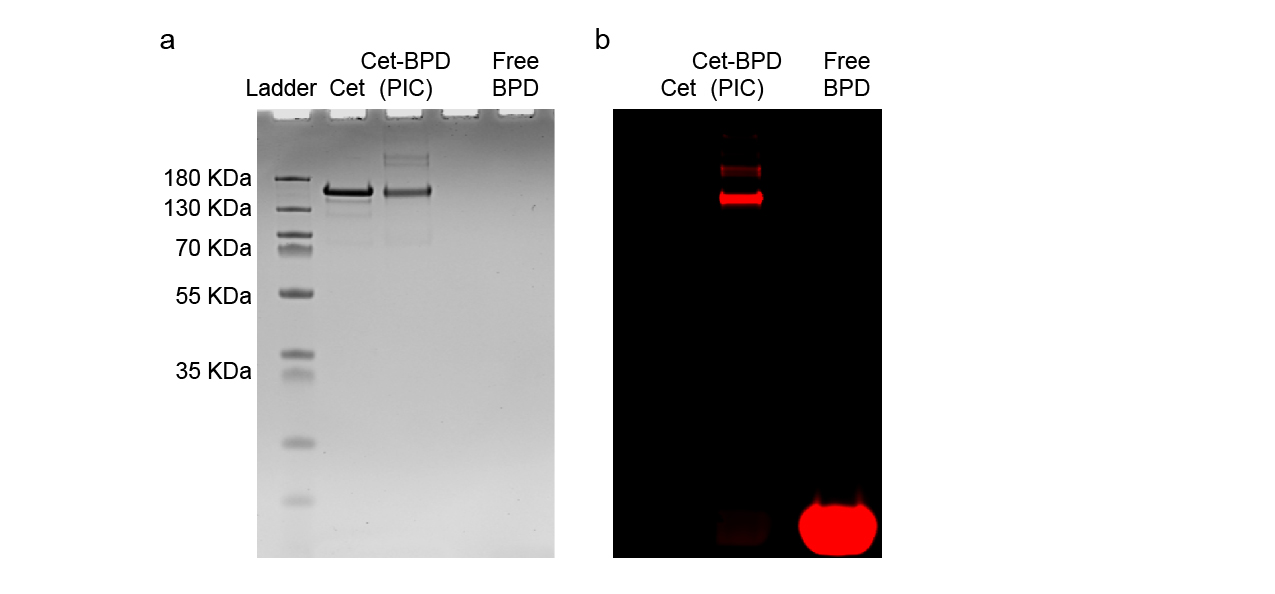


**Figure S5. The purity of Cet-BPD was assessed by gel fluorescence imaging analysis following sodium dodecyl sulfate polyacrylamide gel electrophoresis (SDS-PAGE).** (a) Coomassie blue staining of SDS-PAGE for visualization of the standards (Ladder), Cet, Cet-BPD (PIC), and BPD. (b) Gel fluorescence imaging (Em: 690 nm) of SDS-PAGE shows <1% free BPD impurity in PIC; fluorescence intensity was quantified using ImageJ.
